# Vertical Distribution and Seasonal Patterns of *Candidatus* Nitrotoga in a Sub-Alpine Lake

**DOI:** 10.1264/jsme2.ME23086

**Published:** 2024-05-31

**Authors:** Albin Alfreider, Manuel Harringer

**Affiliations:** 1 Department of Ecology, University of Innsbruck, Technikerstrasse 25, 6020 Innsbruck, Austria

**Keywords:** *Ca.* Nitrotoga, nitrification, limnology, lakes

## Abstract

The nitrite oxidizing bacterial genus *Ca.* Nitrotoga was only recently discovered to be widespread in freshwater systems; however, limited information is currently available on the environmental factors and seasonal effects that influence its distribution in lakes. In a one-year study in a dimictic lake, based on monthly sampling along a vertical profile, the droplet digital PCR quantification of *Ca.* Nitrotoga showed a strong spatio-temporal patchiness. A correlation ana­lysis with environmental parameters revealed that the abundance of *Ca.* Nitrotoga correlated with dissolved oxygen and ammonium, suggesting that the upper hypolimnion of the lake is the preferred habitat.

Nitrification is a central process in the nitrogen cycle that microbially converts ammonia via nitrite to nitrate (NH_3_→NO_2_^–^→NO_3_^–^). The nitrification process is performed by organisms with the ability to use reduced inorganic nitrogen compounds as an energy source and inorganic carbon fixation to reach the carbon demand (Prosser, 1989; Kuypers *et al.*, 2018; Sedlacek, 2020). Research in this field is important to optimize engineered biological nitrogen removal processes, and waste water treatment plants are often employed as model systems to study the niche preferences and environmental adaptations of nitrifying microbes (Daims *et al.*, 2016; Holmes *et al.*, 2019). In contrast to ammonia oxidizers, nitrite oxidizing bacteria (NOB) are poorly characterized in aquatic ecosystems even though this group of microorganisms is widespread in various freshwater systems. Recent studies suggest that Nitrospirae representatives are often the dominant functional guild of NOB in the hypolimnion of deep oligotrophic lakes (Alfreider *et al.*, 2018; Cabello-Yeves *et al.*, 2019; Herber *et al.*, 2020). The recently discovered NOB lineage *Ca.* Nitrotoga, a candidate genus belonging to Betaproteobacteria (*Burkholderiales*), also exhibits a broad habitat range of artificial and natural freshwater systems, including running waters and lakes. *Ca.* Nitrotoga is an important representative of NOB in rivers, being present in the water column and sediments characterized by a wide range of environmental conditions (Fan *et al.*, 2016; Boddicker and Mosier, 2018; Lantz *et al.*, 2021). Previous studies also investigated *Ca.* Nitrotoga in freshwater lakes. Podowski *et al.* (2022) found that *Ca.* Nitrotoga was important in the water column of shallow and productive basins of the Laurentian Great Lakes, whereas *Nitrospirae* dominated deeper and oligotrophic lake types.

An important environmental adaptation of enriched *Ca.* Nitrotoga, which has been extensively examined, is a preference for low temperatures. *Ca.* Nitrotoga is also the only cultured NOB that remains active under cold conditions at 4°C and this group of bacteria is often the dominant NOB in polar ecosystems or permafrost soils (Alawi *et al.*, 2007). The preference of *Ca.* Nitrotoga for cold environments was‍ ‍also confirmed in a lake ecosystem in West Antarctica. In water column samples obtained from subglacial Lake Whillans, a habitat characterized by an average *in situ* temperature of –0.49°C, operational taxonomic units associated with *Ca.* Nitrotoga were highly abundant, accounting for 9–13% of rRNA sequence data (Christner *et al.*, 2014; Achberger *et al.*, 2016).

In the present study, the small dimictic lake Piburger See (PIB) was selected because sequencing studies from previous sampling campaigns detected rRNA gene sequences related to *Ca.* Nitrotoga, while all other NOB, including *Nitrospira* and *Nitrobacter*, were absent (16S rRNA sequence data and sampling information are available in the NCBI BioProject database under accession number PRJNA1034802). The quantification of *amoA* genes specifically targeting Comammox *Nitrospira* from another study also showed that complete ammonia oxidizers were absent or present at negligible amounts in the pelagic zone of this lake (Harringer and Alfreider, 2021). The main objective of the present study was to investigate *Ca.* Nitrotoga in PIB within the framework of a high spatiotemporal resolution in order to examine the vertical distribution and seasonal patterns of this NOB using a sensitive quantitative mole­cular method (droplet digital PCR). These data were analyzed in the context of environmental parameters measured in parallel to identify which factors shape the occurrence of *Ca.* Nitrotoga in the water column of the lake.

PIB is a small freshwater lake located in a crystalline mountain area in Austria (913‍ ‍m‍ ‍a.s.l.; 47°11′42″N, 10°53′20″E). It is characterized by an oligo-mesotrophic nutrient status and a dimictic mixing regime and is generally covered with ice from mid-December until the end of March. The water retention time is approximately two years. More detailed information on the lake is provided elsewhere (Alfreider and Tartarotti, 2019). Samples of the annual cycle in PIB were collected monthly over a period of one year (starting from October 2016) along a vertical profile at 3-m intervals in the deepest part of the lake identified in a previous study (nine samples from depths of 0 to 24 m) (Alfreider and Tartarotti, 2019). The depth profiles of temperature, pH, conductivity, and dissolved oxygen (DO) were measured with a multiparameter probe, and the major cation and anion compositions of the water samples were quantified by ion chromatography as described by Alfreider and Tartarotti (2019).

Microorganisms from lake water (480–1,065‍ ‍mL) were collected without prefiltration on a polyethersulfone filter with a pore size of 0.22‍ ‍μm (Millipore) and stored at –20°C until DNA extraction. Total DNA was extracted using a PowerWater©DNA Isolation Kit (Qiagen) according to the protocol of the manufacturer as described in Alfreider *et al.* (2018) and Alfreider and Tartarotti (2019). Extraction efficiency was assessed by quantifying the recovery of the lysis buffer after the beat-beating step in each sample. These extraction factors were taken into account in the calculation of the ddPCR copy number. Quantitative PCR was performed using a ddPCR system (QX200^TM^; BioRad) following the manufacturer’s instructions. Reactions for ddPCR were prepared using the QX200 ddPCR EvaGreen Supermix (BioRad) with a set-up in 96-well plates according to the protocol of the manufacturer. Two primers sets were evaluated for the quantification of *Ca.* Nitrotoga in PIB, targeting genes coding for 16S rRNA. A primer combination designed by Lücker *et al.* (2015) was tested using samples from two depths (21 and 24 m) obtained from the hypolimnion of the study site in June. These depths were selected because the 16S rRNA sequences analyzed in an unpublished investigation (NCBI database accession number PRJNA1034802) indicated the presence of *Ca.* Nitrotoga at this time and depth in PIB. Only one (21 m) of the two depths produced a PCR product with a very weak signal on the agarose gel; therefore, this primer pair was not used in further ana­lyses. The second primer set tested, Ntg200F/840R, was developed by Alawi *et al.* (2007) for the detection of the genus Nitrotoga in permafrost soils. The primer set has since been successfully used in various environmental and engineered systems, including samples obtained from aquatic environments. (Nowka *et al.*, 2015; Figdore *et al.*, 2018; Ishii *et al.*, 2020; Li *et al.*, 2020; Lantz *et al.*, 2021). At both sampling depths of PIB, 21 and 24 m, the evaluation of the primer combination Ntg200F/840R generated PCR products of the correct length. The optimal annealing temperature for ddPCR was selected from temperature gradient experiments using temperatures between 52–63°C. The highest yield and specificity were obtained at an annealing temperature of 59°C and, thus, the primer set Ntg200F/840R was used at this temperature for all ddPCR reactions.

After droplet generation, PCR plates were heat-sealed (Pierceable Foil Heat Seal; BioRad) and subjected to PCR amplification in a T100 thermal cycler (Biorad). Cycling conditions were as follows: at 95°C for 5‍ ‍min for enzyme activation, followed by 40 cycles of denaturation at 95°C for 30‍ ‍s, primer annealing at 59°C for 30‍ ‍s, and primer extension at 72°C for 1‍ ‍min. Reaction stabilization was achieved by final steps at 4°C for 5‍ ‍min and 90°C for 5‍ ‍min. A ramp rate of 2.5°C s^–1^ was used. In signal measurements, plates were analyzed using the ddPCR reader according to the manufacturer’s instructions. Raw data were further analyzed using QuantaSoft^TM^ Software 1.7.4. (BioRad). Quality control included non-template controls and an examination of the reliability of automated threshold settings by QuantaSoft software.

To further evaluate the PCR primer pair NTG200F/840R for the specific detection of *Ca.* Nitrotoga in PIB lake water samples, a sequence ana­lysis of PCR products obtained from depths of 21 and 24‍ ‍m was performed. The protocol used to prepare clone sequences is described elsewhere (Alfreider *et al.*, 2018). Sanger sequencing of the selected rRNA products was performed by a sequencing service enterprise (Eurofins MWG). Nucleotide sequences were aligned using the MUSCLE algorithm implemented in the MEGA 11 software package (Tamura *et al.*, 2021), followed by the construction of a Neighbor-Joining tree including a bootstrap ana­lysis (1,000 replicates). Spearman’s rank correlation coefficient (rSp) ana­lysis was used to examine the relationships between the abundance of *Ca.* Nitrotoga and selected physicochemical parameters.

PIB showed pronounced seasonal dynamics with stratification patterns characteristic of a dimictic lake. An autumnal mixis was observed by late November 2016, followed by inverse stratification under the ice cover during winter (Fig. S1). In Spring, a thermal overturn was observed shortly after the disappearance of the ice cover at the end of March 2017. Between April and early November, stable summer stratification developed. The surface water temperature peaked at 21.1°C in August (Fig. S1). Based on DO concentrations, both thermal overturns were very short and incomplete, resulting a non-mixing water body (monimolimnion) in the deepest water column of the lake (Fig. S1). This phenomenon was accompanied by significant oxygen depletion in the hypolimnion, where DO saturation decreased to below 10% between June and November, particularly below a depth of 15‍ ‍m (Fig. S1). Conversely, the surface water showed periods of DO supersaturation, reaching a peak of 142% in August at a depth of 6 meters. Nitrate concentrations reached the highest values under ice in the upper hypolimnion (Fig. S2). The depletion of nitrate in the epilimnion of the lake was likely caused by photoautotrophs. In the hypolimnion, a depth-dependent decrease was noted in the concentration of nitrate, which was associated with the marked accumulation of ammonium in the reducing environment at the deepest sampling depths. The highest ammonia concentrations were measured continuously at a depth of 24‍ ‍m and reached a maximum in February at this depth (Fig. S2). Dissolved organic carbon (DOC) measurements showed limited spatio-temporal variability, with concentrations consistently ranging between 1.8–2.8‍ ‍mg L^–1^.

PCR products generated with the NTG200F/840R primer pair were used to analyze 32 clone sequences obtained from the hypolimnion of PIB (Fig. 1). These sequences were mostly identical (20 sequences) or differed at only one or two nucleotide positions, indicating that a phylogenetically very similar group of *Ca.* Nitrotoga was dominant in PIB. *Ca.* Nitrotoga 16S rRNA sequences from PIB were affiliated with *Ca.* Nitrotoga sp. HW29 enriched from a recirculation aquaculture system (Hüpeden *et al.*, 2016) and *Ca.* Nitrotoga sp. 1052 retrieved from permafrost soil (Keuter *et al.*, 2022) as the closest relatives with >99.5% sequence similarity. *Ca.* Nitrotoga arctica (Alawi *et al.*, 2007), also enriched from permafrost soil, several *Ca.* Nitrotoga (LAW, MKT, SPKER; Fig. 1) metagenomes reconstructed from river sediments (Boddicker and Mosier, 2018) and water column samples, and the non-marine cold-adapted *Ca.* Nitrotoga sp. strains AM1 and AMP1 (Ishii *et al.*, 2017, 2020) obtained from coastal sand in an eelgrass zone were the next closest *Ca.* Nitrotoga representatives based on 16S rRNA phylogeny (Fig. 1).

The occurrence of *Ca.* Nitrotoga based on ddPCR data during the annual cycle of PIB showed a very heterogeneous picture. Throughout the year, the highest abundance of *Ca.* Nitrotoga was observed in the hypolimnion of PIB and *Ca.* Nitrotoga showed a pronounced maximum in the upper hypolimnion (a depth of 15 m) during early summer stratification in May (7.95×10^5^ cells L^–1^) (Fig. 2; individual ddPCR measurements are shown in Table S1). During this period and generally during summer stagnation, oxygen conditions in the hypolimnion of PIB were characterized by significant oxygen depletion between May and November at depths below 15‍ ‍m (Fig. S1). The surface water column between a depth of 0 and 10‍ ‍m was characterized by a very low abundance of *Ca.* Nitrotoga throughout the sampling period (Fig. 2). This part of the pelagic zone was also characterized by DO saturation or supersaturation caused by photoautotrophic organisms (Alfreider and Tartarotti, 2019) (Fig. S1). During winter stagnation between mid-December and late March, when large parts of the water column were oxygenated, only a very low abundance of *Ca.* Nitrotoga was detected over the whole water column (Fig. 2). Spearman’s rank correlation ana­lysis with selected environmental parameters (temperature, DO, ammonium, nitrate, and chlo­rophyll a; measurements are shown in Table S2, 3, 4, 5, and 6) revealed correlations with the parameters DO and ammonium concentrations, while temperature showed a negative correlation (Table S7). Nitrate concentrations did not correlate with the spatiotemporal distribution of *Ca.* Nitrotoga in PIB. Since DO and ammonium demonstrated characteristic gradients as a function of depth, Spearman’s correlation ana­lysis also revealed that depth correlated with the occurrence of *Ca.* Nitrotoga (Table S7).

*Ca.* Nitrotoga typically shows high variations in substrate affinity and other physiological properties, and translating culture-based studies to natural habitats remains a challenge. Genomic predictions of *Ca.* Nitrotoga from freshwater habitats indicated that *Ca.* Nitrotoga is metabolically active in the presence of low oxygen or anoxic conditions (Boddicker and Mosier, 2018). This may also explain the negative correlation observed between *Ca.* Nitrotoga and DO in the present study. On the other hand, the higher resistance of *Ca.* Nitrotoga to higher oxygen concentrations than that of *Nitrospira* representatives was proposed in another study based on the genetic inventory and growth on plates of the first pure culture of *Ca.* Nitrotoga fabula isolated from activated sludge (Kitzinger *et al.*, 2018). Similar findings were reported in a study by Zheng *et al.* (2020), in which oxygen deficiency (DO of 1.0–3.0‍ ‍mg O_2_ L^–1^) in activated sludge favored *Nitrospira* when in competition with *Ca.* Nitrotoga. These differences in the adaptation of *Ca.* Nitrotoga and *Nitrospira* may also be explained by the different CO_2_ fixation strategies used by the two NOB groups. The Calvin-Benson-Bassham (CBB) cycle used by *Ca.* Nitrotoga and the reductive tricarboxylic acid (rTCA) cycle found in *Nitrospira* significantly differed in the energy requirements and oxygen tolerance of the pathways (Berg, 2011; Alfreider *et al.*, 2017). Some enzymes in the rTCA cycle are known to be sensitive to oxygen because rTCA-based CO_2_ fixation mostly occurs in anaerobes; however, special modifications by both enzymes to oxic conditions have been reported (Yamamoto *et al.*, 2006; Berg, 2011). If this robustness comes at the expense of a lower specific activity, it may significantly increase the energy demands of the cycle (Berg, 2011). Enzymes involved in the CBB cycle are highly resistant to mole­cular oxygen, giving them a competitive advantage over *Nitrospira* in oxygen-rich environments. (Berg, 2011; Spieck *et al.*, 2021). However, none of the *Ca.* Nitrotoga genomes examined to date have been found to contain genes coding for carboxysomes (Keuter *et al.*, 2022). Without carboxysomes, the CO_2_ fixation potential of *Ca.* Nitrotoga at high oxygen concentrations may be limited by the oxygenase function of the RuBisCO enzyme. It currently remains unclear whether DO concentrations create unique niches for NOB using the rTCA or CCB cycle.

Ammonium concentrations also correlated with *Ca.* Nitrotoga in PIB (Table S7). *Ca.* Nitrotoga has a high tolerance to free ammonia and ammonium with inhibition limits between 25–40‍ ‍mM (Keuter *et al.*, 2022). The closest relatives of *Ca.* Nitrotoga from PIB tolerate ammonium up to concentrations of 25 (strain 1052) and 40 mM (strain HW29) (Hüpeden *et al.*, 2016; Keuter *et al.*, 2022). These values are well above the highest concentrations found in PIB, with a maximum value of 1.239‍ ‍mg L^–1^ (≙ 0.0587‍ ‍mM) NH_4_-N measured in February at a depth of 24‍ ‍m (Table S5 and Fig. S2). Previous studies reported that some strains (*Ca.* Nitrotoga sp. BS and AM1) were stimulated by the addition of ammonium, whereas several other *Ca.* Nitrotoga representatives, including *Ca.* Nitrotoga sp. HW29, did not benefit from the addition of ammonium (Ishii *et al.*, 2017; Wegen *et al.*, 2019). Difficulties are associated with clarifying whether tolerance to ammonium also confers a selective advantage over other NOB and, thus, if ammonium is an important environmental factor that also affects niche differentiation between different groups of NOB because the majority of ammonium inhibition experiments were conducted at markedly higher concentrations than those found in PIB. Furthermore, the strong dependence of the vertical gradients of DO and ammonium concentrations in PIB makes it difficult to identify the individual effects of both parameters on *Ca.* Nitrotoga.

An important environmental adaptation of enriched *Ca.* Nitrotoga, which has been intensively studied, is a preference for low temperatures. *Ca.* Nitrotoga is also the only cultured NOB that remains active under cold conditions at 4°C, and this group of bacteria is often the dominant NOB in polar ecosystems or permafrost soils (Alawi *et al.*, 2007). In the present study, temperature weakly correlated with the abundance of *Ca.* Nitrotoga, with this group being more likely to occur at lower temperatures (Table S7). However, this relationship is hampered by *Ca.* Nitrotoga being restricted to the hypolimnion of PIB, where temperatures are consistently low at between 4 and 8°C. This temperature range does not provide an optimal environment for growth because the temperature optimum of *Ca.* Nitrotoga is typically >10°C depending on the strain (summarized by Keuter *et al.*, 2022). *Ca.* Nitrotoga enrichments 1052 and HW29, which are also the closest relatives based on the 16S rDNA phylogeny of the sequences analyzed from PIB water samples (Fig. 1), both showed optimal growth at 22°C (Keuter *et al.*, 2022). In comparison with other *Ca.* Nitrotoga strains, these temperatures are in the middle range of the temperature optimum for *Ca.* Nitrotoga, with optimal growth being observed at 17°C for other strains, such as *Ca.* Nitrotoga BS and AM-1, and at approximately 25°C for *Ca.* Nitrotoga fabula and *Ca.* Nitrotoga CP45 (Ishii *et al.*, 2017; Kitzinger *et al.*, 2018; Wegen *et al.*, 2019; Lantz *et al.*, 2021, Keuter *et al.*, 2022). Based on these findings, water temperatures in the epilimnion during summer stagnation (maximum temperature of 21.1°C in August) correspond better to the temperature optima observed in *Ca.* Nitrotoga from laboratory studies. To clarify the effects of temperature on the distribution of *Ca.* Nitrotoga in lakes, further studies are needed on different lakes characterized not only by a wide range of temperatures, but also by different oxygen and ammonium gradients

An additional explanation for the absence of *Ca.* Nitrotoga in the surface water of the lake may be photoinhibitory effects. Photosensitivity has been documented for *Ca.* Nitrotoga sp. CP45, but may also impact other *Ca.* Nitrotoga strains as a result of the photooxidation of c-type cytochromes (Lantz *et al.*, 2021). A comparison of Secchi transparency measurements in PIB, which varied between 3.9 and 6‍ ‍m throughout the year, with the occurrence of *Ca.* Nitrotoga in the upper water column (0–9 m) of the lake, showed no relationship between the two parameters (Fig. S3). However, potential light inhibition may also be superimposed by other biological factors, which are particularly important in the euphotic zone and may also affect the environmental range of *Ca.* Nitrotoga. Although the effects of these biological components have not yet been examined in detail, the temporal/spatial distribution of chlo­rophyll a (as an indicator of the presence of photoautotrophic organisms) was not related to the presence of *Ca.* Nitrotoga in the water column of PIB (Table S7). Evidently, the importance of different limnological factors will only be clarified in detail by performing long-term studies on different lakes in combination with *in situ* experiments on the effects of individual parameters on *Ca.* Nitrotoga.

In the present study, we extend current knowledge on the spatiotemporal dynamics of *Ca.* Nitrotoga in freshwater ecosystems. The outcome of this investigation provides a novel insight into the environmental parameters controlling the seasonal and vertical distributions of these NOB in the water column of a lake. The results obtained indicate that *Ca.* Nitrotoga is strongly affected by depth-dependent gradients established during lake stratification, with varying ammonia and dissolved oxygen concentrations being important factors for the occurrence of *Ca.* Nitrotoga.

All sequence data analyzed in the present study have been submitted to the GenBank database under accession numbers MN737203–MN737234.

## Funding

This study was funded by the Austrian Science Fund (Grant-DOI: 10.55776/P36812 and 10.55776/P25703) to Alfreider A.

## Citation

Alfreider, A., and Harringer, M. (2024) Vertical Distribution and Seasonal Patterns of *Candidatus* Nitrotoga in a Sub-Alpine Lake. *Microbes Environ ***39**: ME23086.

https://doi.org/10.1264/jsme2.ME23086

## Supplementary Material

Supplementary Material

## Figures and Tables

**Fig. 1.**
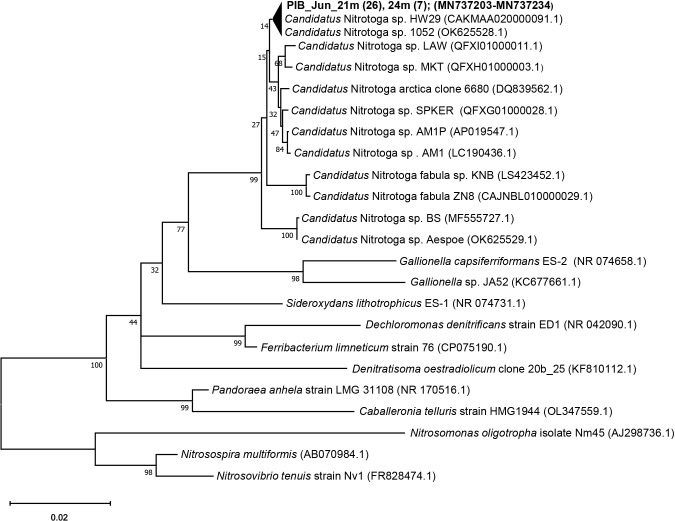
Neighbor-joining phylogenetic tree reflecting relationships between *Ca.* Nitrotoga obtained from lake water samples in this study (shown in bold) and selected relatives obtained from GenBank. Numbers in brackets indicate the number of clones analyzed. Bootstrap values are shown as percentages of 1,000 replicates.

**Fig. 2.**
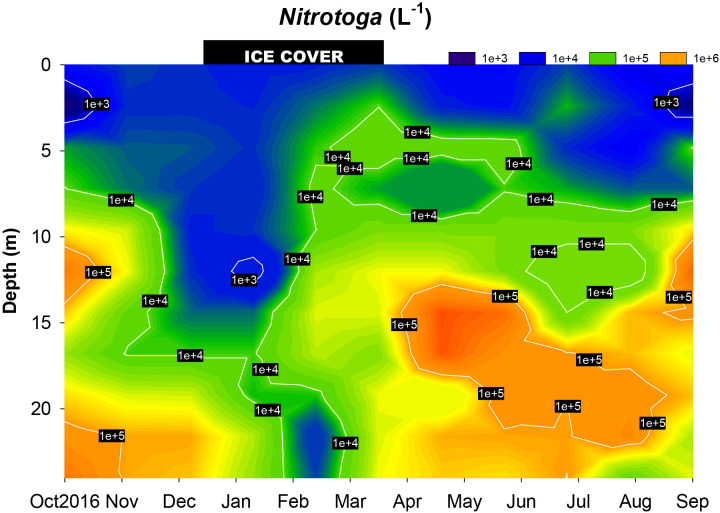
Spatiotemporal dynamics of the abundance of *Ca.* Nitrotoga in water samples collected monthly along vertical profiles (3-m intervals). To calculate the abundance of *Ca.* Nitrotoga, ddPCR measurements (Table S1) were divided by two because *Ca.* Nitrotoga contains two copies of the 16S rRNA gene (Boddicker and Mosier, 2018). Note the logarithmic scale. The black-shaded rectangle at the top of the plot represents the approximate duration of the ice cover.
