# Supplementary material for: Vertical Distribution and Seasonal Patterns of Candidatus Nitrotoga in a Sub-Alpine Lake
Source: Microbes Environ. 2024 May 31;39(2):ME23086. doi: 10.1264/jsme2.ME23086 (PMC11220445; doi:10.1264/jsme2.ME23086)
Supplement: Supplementary file 1 — Supplementary Material [file 39_23086_s1.pdf]

SUPPLEMENTARY MATERIAL

Table S1. Individual ddPCR measurements shown in the contour plot in the main text (Fig 2).

**Depth [m]**	**Month**	***Ca.* Nitrotoga *16S rDNA* [10^3 Copies L^{-1}]**	**Month**	***Ca.* Nitrotoga *16S rDNA* [10^3 Copies L^{-1}]**	**Month**	***Ca.* Nitrotoga *16S rDNA* [10^3 Copies L^{-1}]**
0	October 2016	4.2	February	5.2	June	5.1
3		2.6		6.0		4.2
6		4.8		7.5		n.d.
9		23.9		8.3		5.6
12		479.5		6.9		13.5
15		72.7		5.2		127.0
18		106.6		5.7		297.0
21		384.4		7.5		197.3
24		553.3		21.6		152.8
0	November	7.8	March	6.9	July	8.0
3		7.2		25.8		15.8
6		7.5		38.4		7.0
9		59.3		28.4		2.5
12		119.6		128.9		3.8
15		21.5		117.7		130.8
18		45.1		61.4		201.3
21		167.1		29.2		206.4
24		194.3		24.2		191.2
0	December	6.6	April	6.7	August	2.1
3		6.9		11.1		4.0
6		9.1		4.4		4.6
9		9.0		11.9		3.2
12		8.6		24.8		5.6
15		6.6		47.5		179.1
18		10.1		244.7		196.7
21		185.3		126.0		344.3
24		170.0		160.2		15.0
0	January	5.4	May	2.6	September 2017	4.1
3		7.3		4.0		4.0
6		8.4		2.3		4.0
9		7.8		6.0		5.3
12		9.0		15.8		701.1
15		6.8		1590		120.4
18		3.9		184.1		158.0
21		7.2		186.4		80.7
24		73.6		124.6		85.7

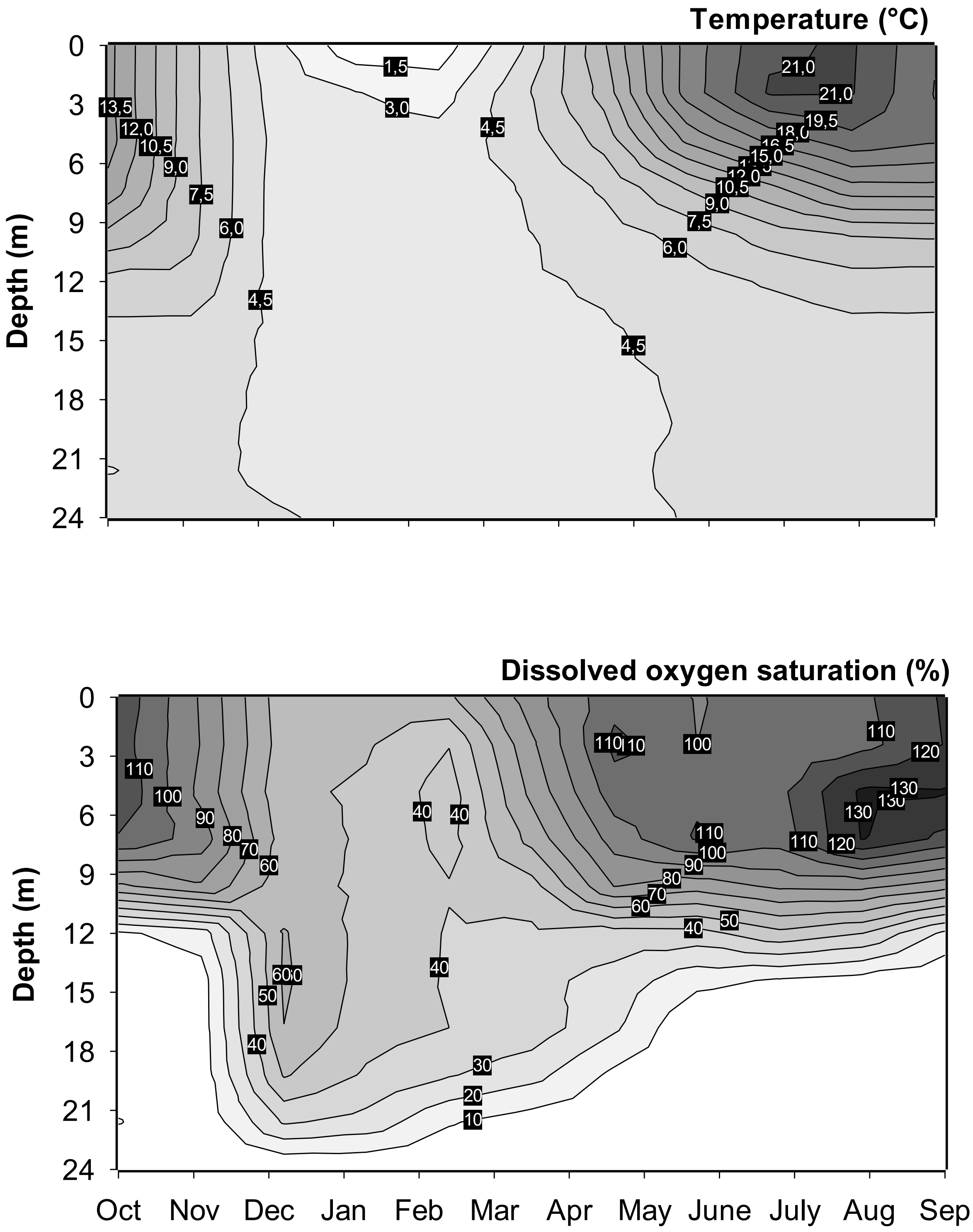

Fig S1. Spatiotemporal dynamics of temperature and dissolved oxygen measured on a monthly basis over a period of one year.

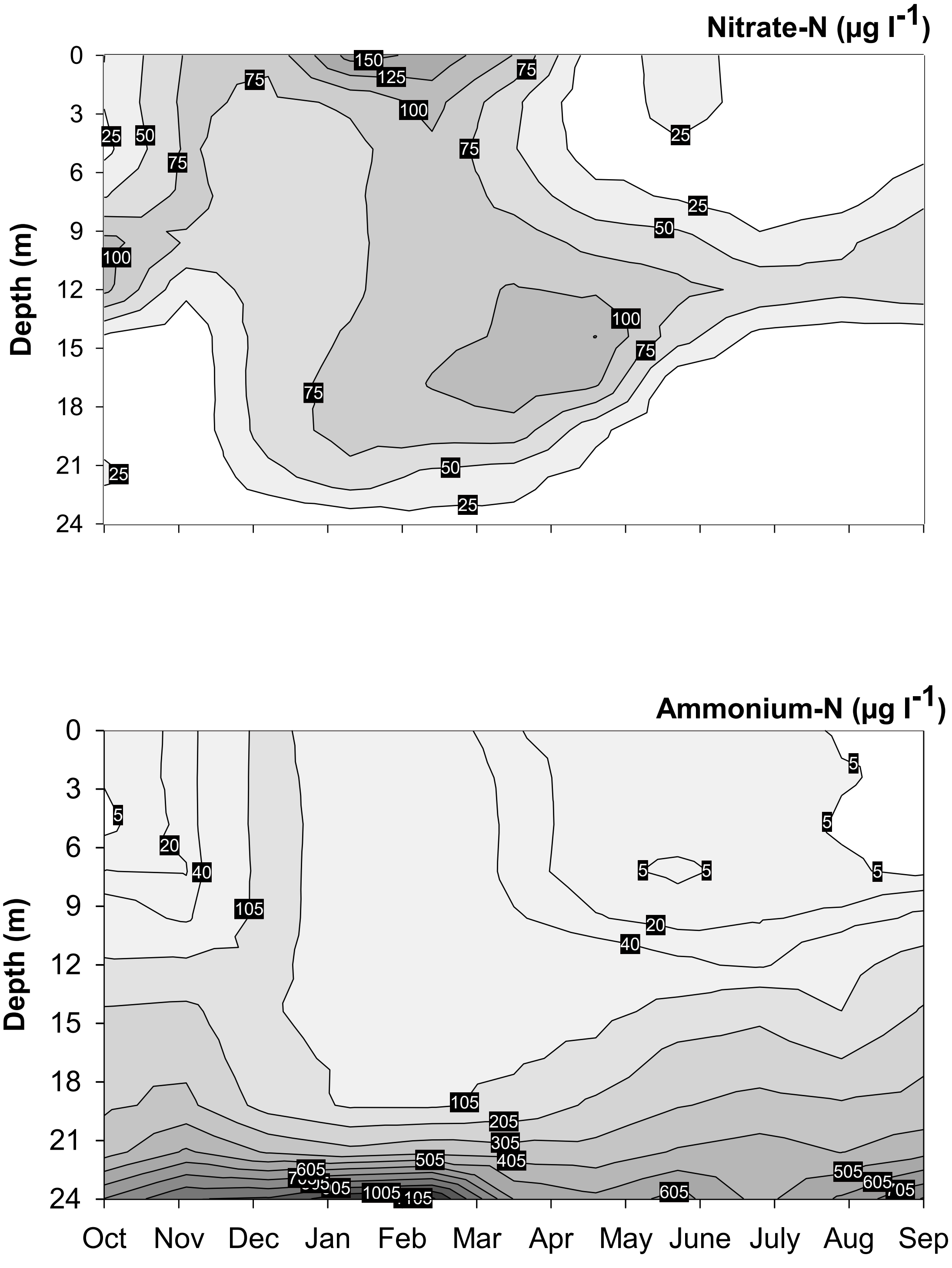

Fig. S2. Spatio-temporal dynamics of nitrate and ammonium measured on a monthly basis over a period of one year.

Table S2. Temperature measurements

Depth [m]	Month	Temperature [°C]	Month	Temperature [°C]	Month	Temperature [°C]
0	October 2016	14	February	1.1	June	19.1
3		14		2.6		18.8
6		13.95		3.9		12.2
9		10.9		4.05		7.4
12		6.95		4.05		5.6
15		5.5		4.1		4.85
18		5		4.1		4.6
21		4.8		4.2		4.7
24		4.8		4.4		4.55
0	November	8.4	March	1	July	20.8
3		8.4		3.8		20.7
6		8.4		4.05		14.5
9		8.4		4.1		9.3
12		7.2		4.15		6.3
15		5.5		4.2		5
18		4.95		4.2		4.8
21		4.7		4.25		4.6
24		4.7		4.4		4.55
0	December	4.4	April	8.15	August	21.1
3		4.5		7.4		20.7
6		4.4		5.7		17.4
9		4.4		4.85		10.2
12		4.4		4.5		6.9
15		4.4		4.35		5.4
18		4.4		4.35		4.9
21		4.4		4.35		4.7
24		4.6		4.4		4.7
0	January	0.5	May	11.8	September 2017	18.1
3		3.6		11.2		18
6		3.9		8.35		17.2
9		3.9		6.05		10.3
12		4		4.8		6.7
15		4.05		4.45		5.45
18		4.1		4.4		4.9
21		4.15		4.35		4.75
24		4.4		4.4		4.7

Table S3. Dissolved oxygen measurements

Depth [m]	Month	Dissolved oxygen [mg L^{-1}]	Month	Dissolved oxygen [mg L^{-1}]	Month	Dissolved oxygen [mg L^{-1}]
0	October 2016	10.77	February	7.56	June	8.40
3		10.58		5.12		8.45
6		10.86		4.81		10.68
9		7.97		4.87		8.73
12		0.89		4.79		4.36
15		0.17		4.76		0.47
18		0		4.55		0
21		0		2.37		0
24		0		0.32		0
0	November	9.63	March	7.90	July	8.60
3		9.71		5.30		8.60
6		9.59		5.00		10.60
9		9.77		5.10		10.00
12		1.22		4.40		5.30
15		0.08		4.30		0.40
18		0		4.20		0
21		0		1.00		0
24		0		0.00		0
0	December	6.68	April	11.40	August	8.63
3		6.71		11.70		8.86
6		6.50		10.10		12.15
9		6.60		7.00		9.42
12		6.63		4.40		4.18
15		6.65		3.50		0.14
18		6.23		3.20		0.13
21		3.91		0.80		0
24		0		0.00		0
0	January	6.43	May	9.60	September 2017	10.21
3		6.02		10.20		10.37
6		5.94		11.10		10.96
9		5.91		10.80		8.81
12		5.99		3.90		1.92
15		5.97		2.50		0.11
18		5.39		0.60		0
21		2.56		0.40		0
24		0		0		0

Table S4. Nitrate measurements

Depth [m]	Month	Nitrate-N [µg L^{-1}]	Month	Nitrate-N [µg L^{-1}]	Month	Nitrate-N [µg L^{-1}]
0	October 2016	0	February	122	June	34
3		25		88		34
6		23		81		10
9		98		82		49
12		107		86		77
15		5		89		19
18		0		97		0
21		0		70		0
24		0		15		0
0	November	79	March	166	July	7
3		82		100		7
6		80		93		5
9		80		95		17
12		32		98		68
15		0		103		6
18		0		106		7
21		0		32		7
24		0		16		8
0	December	64	April	19	August	0
3		62		0		0
6		58		26		7
9		62		72		39
12		59		102		57
15		58		110		0
18		60		117		0
21		41		45		0
24		0		16		0
0	January	165	May	27	September 2017	5
3		67		23		0
6		67		19		31
9		67		53		61
12		67		92		68
15		75		121		0
18		78		18		0
21		65		0		0
24		0		0		0

Tab. S5. Ammonium measurements

Depth [m]	Month	Ammonium-N [µg L^{-1}]	Month	Ammonium-N [µg L^{-1}]	Month	Ammonium-N [µg L^{-1}]
0	October 2016	7	February	58	June	8
3		5		76		8
6		4		81		3
9		53		81		4
12		113		85		40
15		243		89		189
18		307		91		278
21		443		219		389
24		708		1239		625
0	November	15	March	44	July	12
3		14		62		21
6		14		65		9
9		14		66		21
12		113		88		37
15		256		93		203
18		304		100		283
21		456		294		423
24		893		805		457
0	December	130	April	5	August	2
3		131		2		4
6		130		4		3
9		130		16		18
12		129		67		84
15		131		91		116
18		159		106		271
21		326		270		373
24		918		504		645
0	January	58	May	21	September 2017	3
3		75		21		1
6		77		16		0
9		75		14		27
12		73		63		144
15		85		96		235
18		99		233		313
21		181		333		413
24		996		583		813

Table S6. Chlorophyll a measurements

Depth [m]	Month	Chlorophyll a [µg L^{-1}]	Month	Chlorophyll a [µg L-1]	Month	Chlorophyll a [µg L^{-1}]
0	October 2016	2.75	February	4.72	June	2.42
3		3.01		3.01		1.70
6		3.01		2.55		2.62
9		2.10		2.16		5.63
12		5.24		1.24		6.48
15		4.78		0.79		3.99
18		3.60		0.79		5.57
21		2.36		1.18		3.14
24		2.36		2.42		3.73
0	November	2.03	March	1.70	July	3.01
3		2.36		5.63		3.01
6		2.16		7.07		4.06
9		2.10		6.61		4.26
12		7.47		2.16		3.60
15		2.88		0.79		5.30
18		2.55		0.59		4.45
21		1.57		0.72		2.55
24		1.83		1.05		2.62
0	December	1.38	April	9.56	August	2.16
3		1.64		19.71		1.90
6		1.44		20.96		2.36
9		1.44		13.49		3.60
12		1.51		6.61		2.55
15		1.64		4.19		9.96
18		1.50		2.88		5.37
21		1.57		2.69		2.03
24		2.10		3.67		2.10
0	January	29.14	May	0.65	September 2017	3.21
3		5.96		0.98		3.34
6		2.23		1.51		2.82
9		2.42		5.24		3.47
12		1.57		4.39		1.64
15		1.18		2.62		6.75
18		1.05		1.70		3.21
21		1.38		1.70		1.83
24		2.42		5.63		1.96

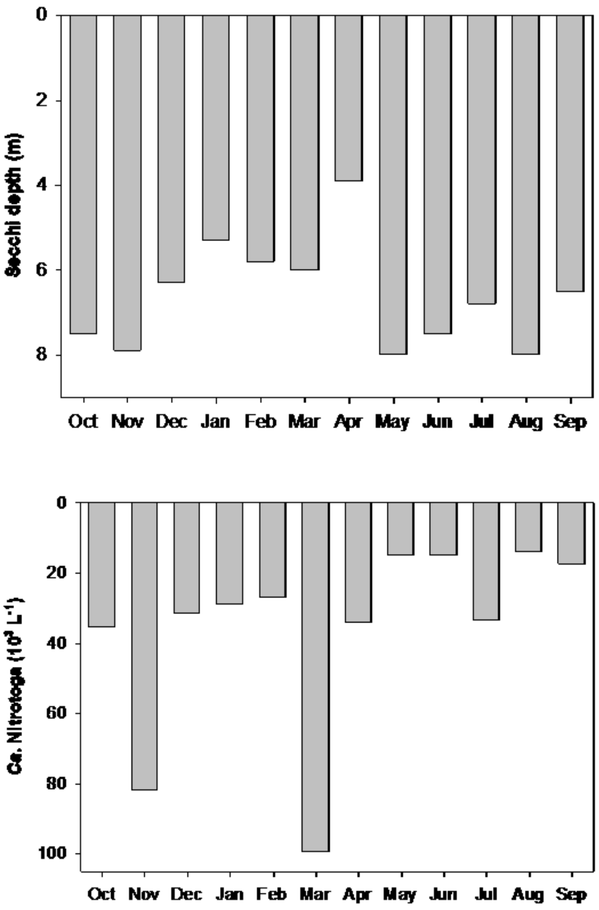

Fig. S3. Seasonal dynamics of Secchi depths and the sum of *Ca.* Nitrotoga abundances from sampling depths 0 to 9m.

Table S7. Spearman rank correlation comparing selected environmental parameters and *Ca*. Nitrotoga abundances

Variable	Depth	Temperature	Oxygen	NO_3-N	NH_4-N	Chlorophyll a
Temperature	-0.39**					
Oxygen	-0.85**	0.41**				
NO_3-N	-0.34**	-0.44**	0.33**			
NH_4-N	0.87**	-0.47**	-0.92**	-0.34**		
Chlorophyll a	-0.17	0.21	0.01	-0.12	-0.20	
Ca. Nitrotoga	0.68**	-0.28*	-0.80**	-0.21	0.75**	0.11

Significant at *p<0.01; **p<0.001
